# Supplementary material for: Rab10 inactivation promotes AMPAR trafficking and spine enlargement during long-term potentiation
Source: bioRxiv. 2025 Aug 27:2022.05.17.492345. Preprint. [Version 6] doi: 10.1101/2022.05.17.492345 (PMC12154598; doi:10.1101/2022.05.17.492345)
Supplement: Supplement 1 [file NIHPP2022.05.17.492345v6-supplement-1.pdf]

bioRxiv preprint doi: https://doi.org/10.1101/2022.05.17.492345; this version posted August 28, 2025. The copyright holder for this preprint (which was not certified by peer review) is the author/funder, who has granted bioRxiv a license to display the preprint in perpetuity. It is made available under aCC-BY 4.0 International license.

Figure Supplement

Rab10 inactivation promotes AMPAR trafficking and spine enlargement during long-term potentiation

Jie Wang[1,2,6], Jun Nishiyama[2,4], Paula Parra-Bueno[2], Elwy Okaz[2], Goksu Oz[2], Xiaodan Liu[2], Tetsuya Watabe[2], Irena Suponitsky-Kroyter[2], Timothy E McGraw[3], Erzsebet M. Szatmari[2,5], Ryohei Yasuda[2,*]

1. Department of Neurobiology, Duke University School of Medicine, Durham, NC, 27710, USA

2. Neuronal Signal Transduction Group, Max Planck Florida Institute for Neuroscience, Jupiter, FL, 33458, USA

3. Weill Cornell Medicine Graduate School of Medical Sciences, 1300 York Ave. Box 65 New York, NY 10065

4. Current Address: Program in Neuroscience and Behavioral Disorders, Duke-NUS Medical School, 8 College Road, Singapore 169857, Singapore.

5. Current Address: Department of Physical Therapy, East Carolina University, Greenville NC, USA.

6. Current Address: Division of Life Science, The Hong Kong University of Science and Technology, Hong Kong, China

*Correspondence to: ryohei.yasuda@mpfi.org

bioRxiv preprint doi: https://doi.org/10.1101/2022.05.17.492345; this version posted August 28, 2025. The copyright holder for this preprint (which was not certified by peer review) is the author/funder, who has granted bioRxiv a license to display the preprint in perpetuity. It is made available under aCC-BY 4.0 International license.

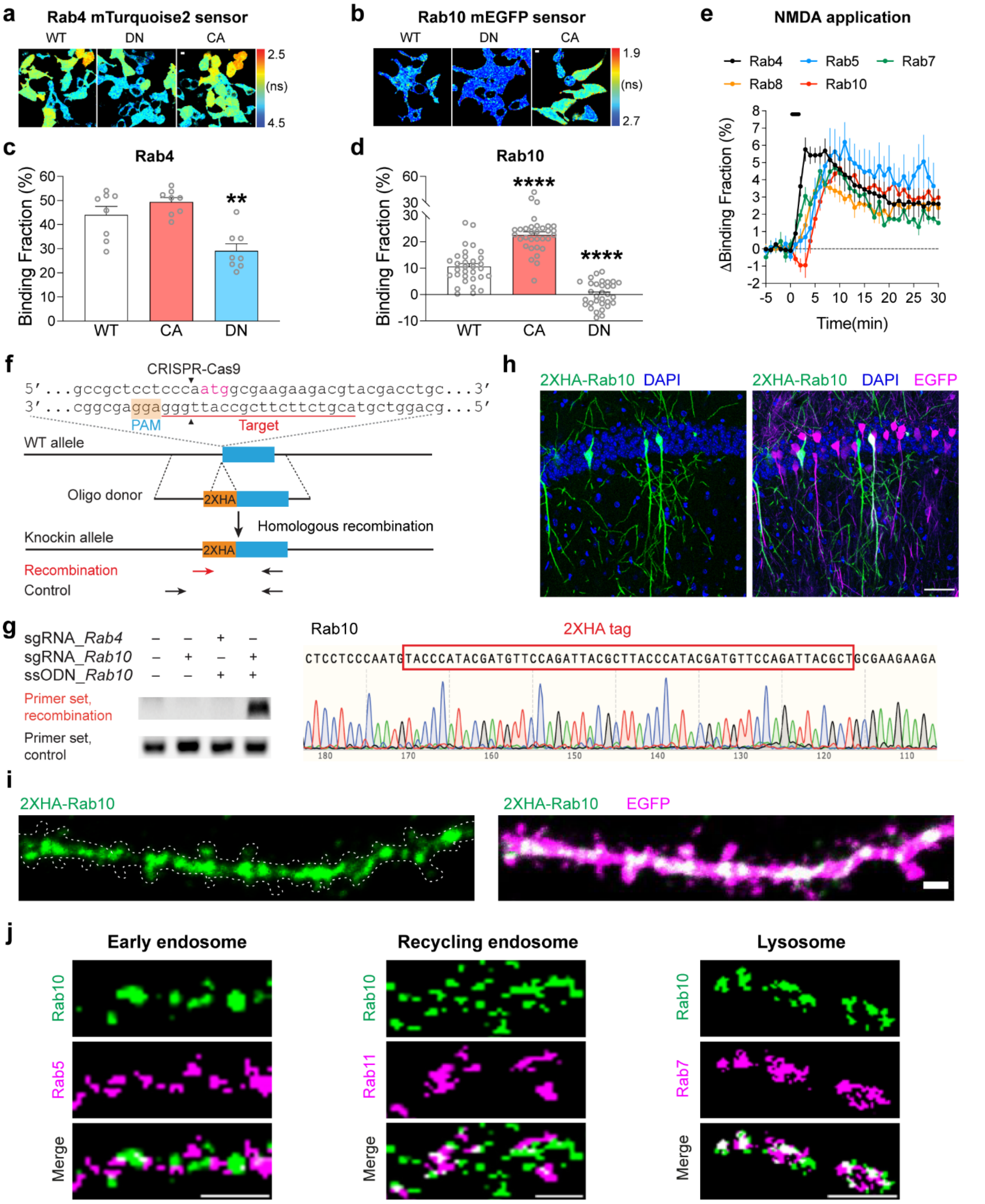

Figure 1-figure supplement 1 | mTurquoise2-Rab4 and mEGFP-Rab10 FRET sensors in HEK 293T Cells, all Rab sensor activity changes upon NMDA application, and localization of endogenous Rab10.

bioRxiv preprint doi: https://doi.org/10.1101/2022.05.17.492345; this version posted August 28, 2025. The copyright holder for this preprint (which was not certified by peer review) is the author/funder, who has granted bioRxiv a license to display the preprint in perpetuity. It is made available under aCC-BY 4.0 International license.

(**a**) Representative fluorescence lifetime images of HEK 293T cells transfected with mTurquoise2-Rab4 sensors. Scale bars represent 5 µm. (**b**) Representative fluorescence lifetime images of HEK 293T cells transfected with mEGFP-Rab10 sensors. Scale bars represent 5 µm. (**c**) Binding fraction of mTurquoise2-Rab4 sensors. Data represent mean ± SEM (** $p<0.01$, one-way ANOVA followed by Bonferroni's multiple comparison tests). N=8, 8, and 8 from left to right. (**d**) Binding fraction of mEGFP-Rab10 sensors. Data represent mean ± SEM (**** $p<0.0001$, one-way ANOVA followed by Bonferroni's multiple comparison tests). N=32, 32, and 32 from left to right. **(e)** Averaged time courses of Rab sensor binding fraction change by NMDA application in rat hippocampal CA1 pyramidal neurons. Data represent mean ± SEM. N=4-11 for each group. **(f)** Schematics of HA-tag knockin into endogenous Rab10 by SLENDR technique. Mouse genomic loci of Rab10 shows the target sites for Cas9, sgRNA and ssODNs. The sgRNA target regions and PAM sequences are labelled with red and orange, respectively. The start codons are marked with magenta. The Cas9 cleavage sites are indicated by black arrowheads. The recombination and control primer sets are in arrows. **(g)** Validation of SLENDR-mediated 2XHA tag insertion into endogenous Rab10. Left: PCR genotyping in genomic DNA extracted from Neuro 2a cells electroporated with indicated sgRNA and ssODNs. Right: Sanger sequencing demonstrated the knockin of 2XHA tag to the N-terminus of endogenous Rab10. **(h)** Confocal microscopic images of the hippocampal CA1 region at P37 stained with DAPI (blue), HA tag fused to the N-terminus of endogenous Rab10 (green) and EGFP (magenta). Scale bar is 50 µm. **(i)** Representative images of the secondary apical dendrites of CA1 pyramidal neurons stained with HA tag fused to the N-terminus of endogenous Rab10 (green) and EGFP (magenta). Scales bar is 1 µm. **(j)** Representative images of endogenous Rab10 (green, SLENDR-mediated 2XHA tag knockin) and exogenous endosomal markers (magenta) in dendrites of CA1 pyramidal neurons. Exogenously expressed mEGFP-Rab5a, mCherry-Rab11a and mEGFP-Rab7 were used as markers for early endosome, recycling endosome and lysosome, respectively. Scales bars are 1 µm.

bioRxiv preprint doi: https://doi.org/10.1101/2022.05.17.492345; this version posted August 28, 2025. The copyright holder for this preprint (which was not certified by peer review) is the author/funder, who has granted bioRxiv a license to display the preprint in perpetuity. It is made available under aCC-BY 4.0 International license.

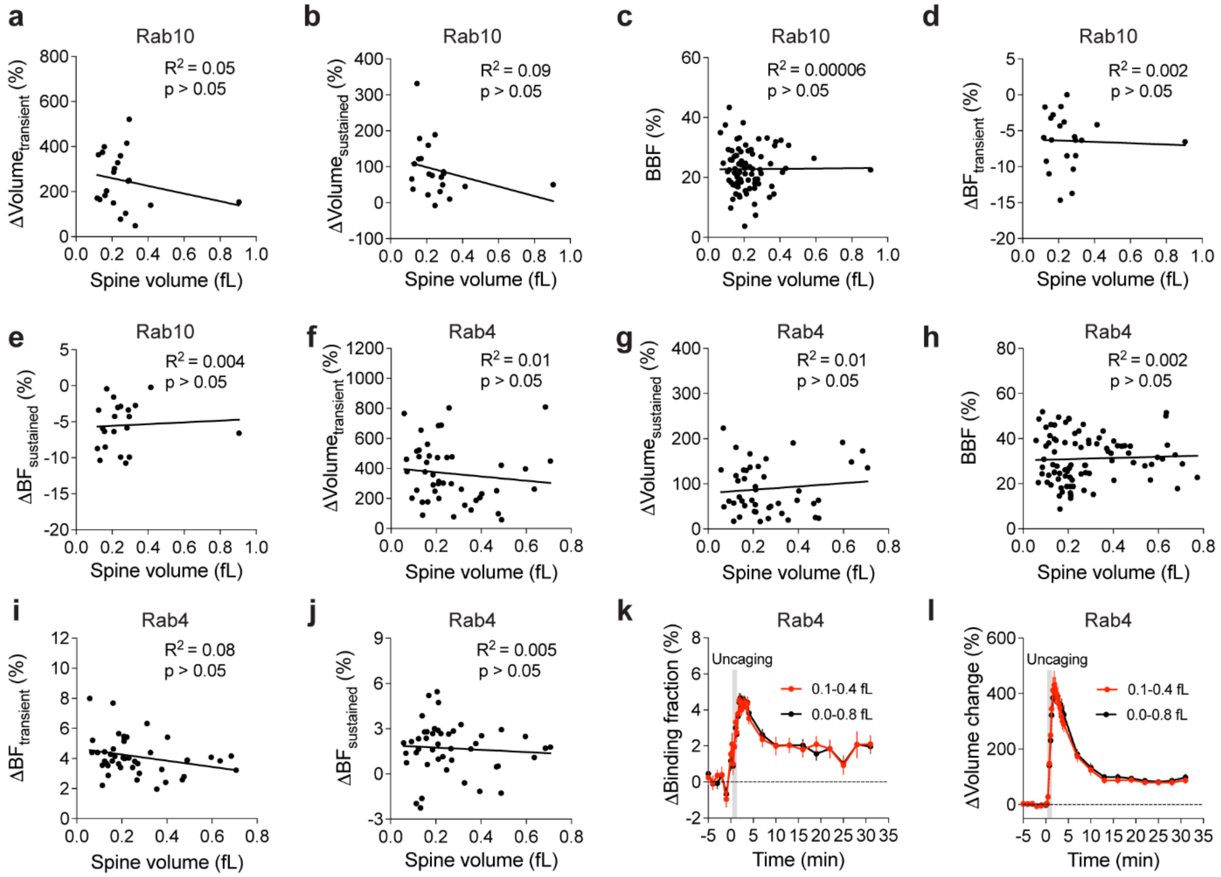

Figure 2-figure supplement 1| Relationship between initial spine volume and basal Rab GTPase activity, spine volume change or activity change during sLTP.

(a and b) Relationship between the initial spine volume and spine volume changes during the transient (a, averaged over 1.3-4 min) or sustained phase (b, averaged over 19-31 min) in neurons expressing Rab10 sensor. N=21. No significant correlation ($p > 0.05$) was found. **(c)** Relationship between spine volume and basal binding fraction (BBF) of Rab10 sensor. N=81. No significant correlation ($p > 0.05$) was found. **(d and e)** Relationship between the initial spine volume and changes in binding fraction of Rab10 sensor during the transient (d, averaged over 1.3-4 min) or sustained phase (e, averaged over 19-31 min) of sLTP in the stimulated spines. N=21. No significant correlation ($p > 0.05$) was found. **(f and g)** Relationship between the initial spine volume and spine volume changes during the transient (f, averaged over 1.3-4 min) or sustained phase (g, averaged over 19-31 min) in neurons expressing Rab4 sensor. N=45. No significant correlation ($p > 0.05$) was found. **(h)** Relationship between spine volume and basal binding fraction (BBF) of Rab4 sensor. N=90. No significant correlation ($p > 0.05$) was found. **(i and j)** Relationship between the initial spine volume and Rab4 activity changes during the transient (i, averaged over 1.3-4 min) or sustained phase (j, averaged over 19-31 min) of sLTP in the stimulated spines. N=45. No significant correlation ($p > 0.05$) was found. **(k)** Time courses of binding fraction changes for Rab4 sensor-expressing spines with initial volumes

bioRxiv preprint doi: https://doi.org/10.1101/2022.05.17.492345; this version posted August 28, 2025. The copyright holder for this preprint (which was not certified by peer review) is the author/funder, who has granted bioRxiv a license to display the preprint in perpetuity. It is made available under aCC-BY 4.0 International license.

ranging from 0.1 fL to 0.4 fL (red, N=32) and 0.0 to 0.8 fL (black, N=45). Data are presented as mean ± SEM. **(l)** Time courses of volume changes for Rab4 sensor-expressing spines with initial volumes ranging from 0.1 fL to 0.4 fL (red, N=32) and 0.0 to 0.8 fL (black, N=45). Data are presented as mean ± SEM.

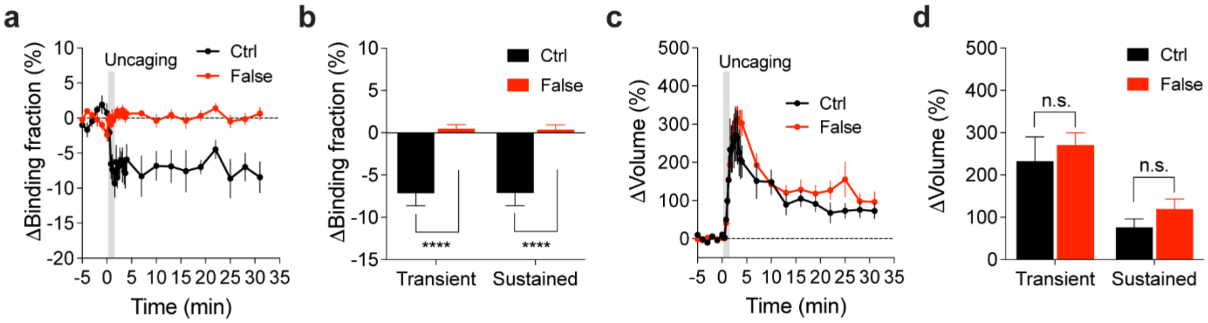

Figure 2-figure supplement 2 | Binding fraction changes of mTurquoise2-Rab10 paired with false acceptor during sLTP.

(a) Averaged time course of changes in binding fraction of Rab10 sensor (Ctrl, black) in the stimulated spines during sLTP. When mTurquoise2-Rab10 was paired with a false acceptor (False, red), mVenus-Rabenosyn5 [439-503]-mVenus, little activity change was observed. Data represent mean ± SEM. N=7/7 and 14/9 (spine/neuron) for Ctrl and False, respectively. **(b)** Quantification of changes in binding fraction during the transient phase (1.3-4 min) and sustained phase (19-31 min) for the same experiments as in (a). Data represent mean ± SEM (**** p<0.0001, Student's t-tests). **(c)** Averaged time courses of changes in spine volume for the same experiments as in (a). Data represent mean ± SEM. **(d)** Quantification of changes in spine volume during the transient phase (1.3-4 min) and sustained phase (19-31 min) for the same experiments as in (a). Data represent mean ± SEM (n.s., not significant, Student's t-tests). Please note the quantification data in (b) and (d) are also presented in Figure 2c,d,f,g.

bioRxiv preprint doi: https://doi.org/10.1101/2022.05.17.492345; this version posted August 28, 2025. The copyright holder for this preprint (which was not certified by peer review) is the author/funder, who has granted bioRxiv a license to display the preprint in perpetuity. It is made available under aCC-BY 4.0 International license.

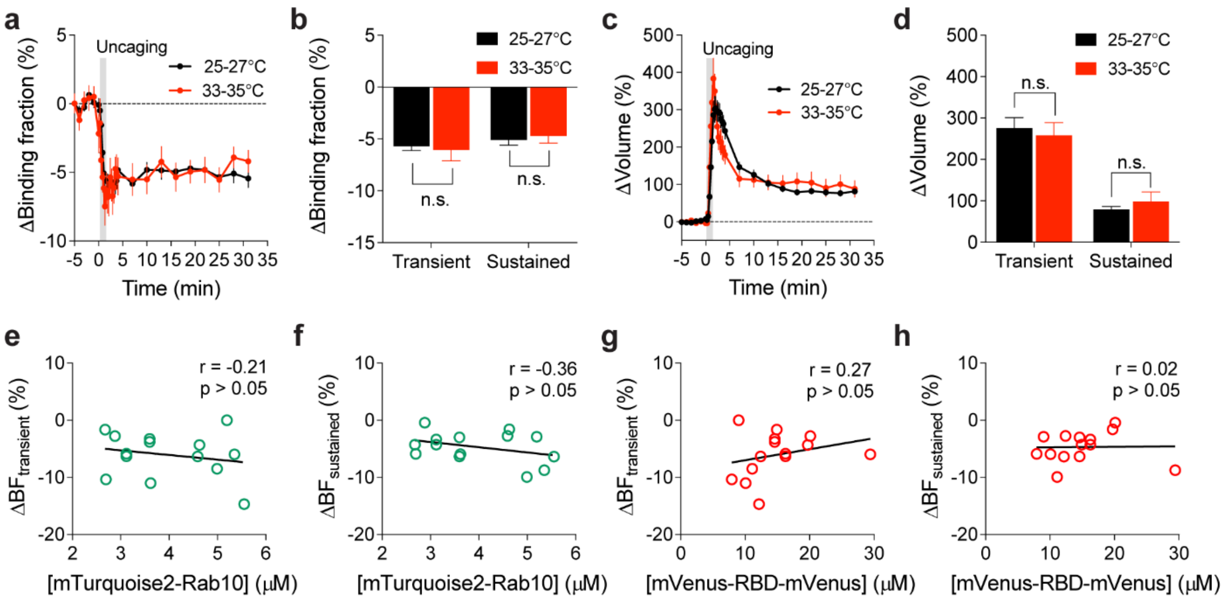

Figure 2-figure supplement 3 | Inactivation of Rab10 during sLTP induced at near physiological temperature.

(a) Averaged time courses of changes in binding fraction of Rab10 sensor in the stimulated spines during sLTP at 25-27°C (black) and 33-35°C (red). Data represent mean ± SEM. N=49/42 and 14/12 (spine/neuron) for 25-27°C and 33-35°C, respectively. **(b)** Quantification of changes in binding fraction in the transient phase (1.3-4 min) and sustained phase (19-31 min) for the same experiments as in (a). Data represent mean ± SEM (n.s., not significant, Student's t-tests). **(c)** Averaged time courses of changes in spine volume for the same experiments as in (a). Data represent mean ± SEM. **(d)** Quantification of changes in spine volume during the transient phase (1.3-4 min) and sustained phase (19-31 min) for the same experiments as in (a). Data represent mean ± SEM (n.s., not significant, Student's t-tests). Please note that the 25-27°C (black) samples in (a-d) are the same as those in Figure 2b, e. **(e and f)** Relationship between mTurquoise2-Rab10 concentration and changes in binding fraction during the transient (e, 1.3-4 min) and sustained phase (f, 19-31 min) of sLTP at 33-35°C. N=14/12 (spine/neuron). No significant correlation ($p > 0.05$) was found. **(g and h)** Relationship between mVenus-RBD-mVenus concentration and changes in binding fraction during the transient (g, 1.3-4 min) and sustained phase (h, 19-31 min) of sLTP at 33-35°C. N=14/12 (spine/neuron). No significant correlation ($p > 0.05$) was found.

bioRxiv preprint doi: https://doi.org/10.1101/2022.05.17.492345; this version posted August 28, 2025. The copyright holder for this preprint (which was not certified by peer review) is the author/funder, who has granted bioRxiv a license to display the preprint in perpetuity. It is made available under aCC-BY 4.0 International license.

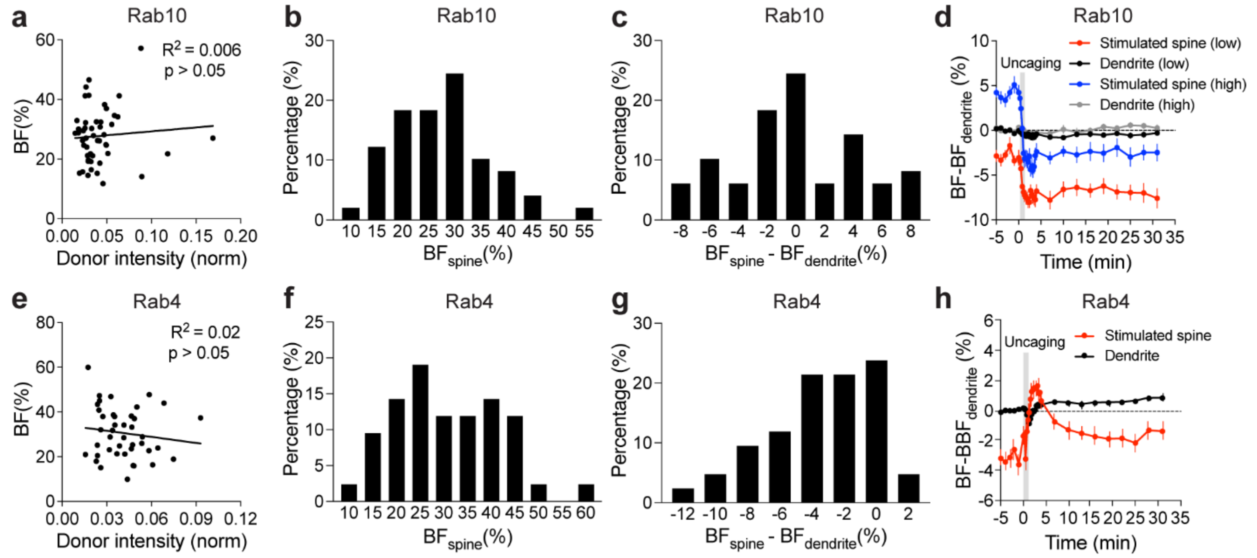

Figure 2-figure supplement 4 | Properties of basal binding fraction and binding fraction change in the stimulated spines for Rab10 and Rab4 sensors.

(a) Relationship between normalized donor intensity and spine binding fraction (BF) for Rab10 sensor. N=49/42 (spine/neuron). No significant correlation ($p > 0.05$) was found. **(b)** Frequency distribution of basal binding fraction in the stimulated spines (BF_{spine}) for Rab10 sensor. N=49/42 (spine/neuron). **(c)** Frequency distribution of basal binding fraction difference between the spines and dendrite (BF_{spine} - $BF_{dendrite}$) for Rab10 sensor. N=49/42 (spine/neuron). **(d)** Averaged time course of binding fraction (BF) subtracted by basal binding fraction (BBF) of dendrite for the stimulated spines and dendrite in Rab10 sensor expressing neurons. For spines with a higher BF (high, blue) than the dendrite (high, gray), Rab10 activity decreased to a level lower than that of the dendrite during sLTP. Data represent mean ± SEM. N=21/18 (spine/neuron). For spines with a lower BF (low, red) than the dendrite (low, black), Rab10 activity still decreased during sLTP. Data represent mean ± SEM. N=28/24 (spine/neuron). **(e)** Relationship between normalized donor intensity and spine binding fraction for Rab4 sensor. N=42/34 (spine/neuron). No significant correlation ($p > 0.05$) was found. **(f)** Frequency distribution of basal binding fraction in the stimulated spines for Rab4 sensor. N=42/34 (spine/neuron). **(g)** Frequency distribution of basal binding fraction difference between the spines and dendrite for Rab4 sensor. N=42/34 (spine/neuron). **(h)** Averaged time course of binding fraction subtracted by basal binding fraction of dendrite for the stimulated spines and dendrite in Rab4 sensor expressing neurons. Data represent mean ± SEM. N=42/34 (spine/neuron).

bioRxiv preprint doi: https://doi.org/10.1101/2022.05.17.492345; this version posted August 28, 2025. The copyright holder for this preprint (which was not certified by peer review) is the author/funder, who has granted bioRxiv a license to display the preprint in perpetuity. It is made available under aCC-BY 4.0 International license.

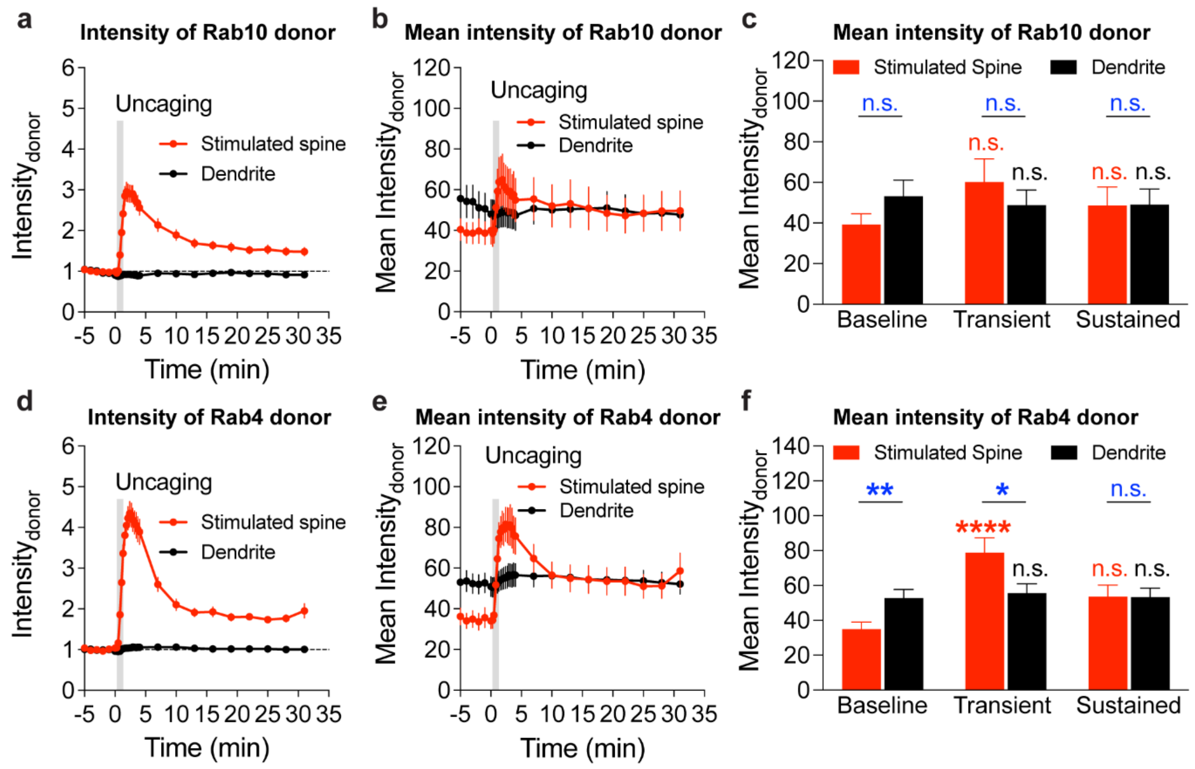

Figure 2-figure supplement 5 | Intensity and mean intensity of Rab10 and Rab4 sensor donors in the stimulated spines and dendrites during sLTP.
(a and b) Averaged time courses of Rab10 donor intensity (a) and mean intensity (b) in the stimulated spines (red) and dendrites (black) during sLTP. Data represent means ± SEM. N=49/42 (spine/neuron) and 49/42 (dendrite/neuron) for the stimulated spine and dendrite, respectively. **(c)** Quantification of Rab10 donor mean intensity in the baseline (averaged over -5-0 min), transient phase (averaged over 1.3-4 min) and sustained phase (averaged over 19-31 min) for the experiments in (b). Data represent means ± SEM. Red color statistics indicate comparisons with baseline in the stimulated spines, and black color statistics indicate comparisons with baseline in the dendrites (n.s., not significant, two-way ANOVA). Blue color statistics indicate comparisons between the stimulated spines and dendrites (n.s., not significant, Student's t-tests). **(d and e)** Averaged time courses of Rab4 donor intensity (d) and mean intensity (e) in the stimulated spine (red) and dendrite (black) during sLTP. N=42/34 (spine/neuron) and 42/34 (dendrite/neuron) for the stimulated spine and dendrite, respectively. **(f)** Quantification of Rab4 donor mean intensity in the baseline (averaged over -5-0 min), transient phase (averaged over 1.3-4 min) and sustained phase (averaged over 19-31 min) for the experiments in (e). Data represent means ± SEM. Red color statistics indicate comparisons with baseline in the stimulated spines and black color statistics indicate comparisons with baseline in the dendrites (n.s., not significant, **** p<0.0001, two-way ANOVA). Blue color statistics indicate comparisons between the stimulated spines and dendrites (n.s., not significant, * p<0.05, ** p<0.01, Student's t-tests).

bioRxiv preprint doi: https://doi.org/10.1101/2022.05.17.492345; this version posted August 28, 2025. The copyright holder for this preprint (which was not certified by peer review) is the author/funder, who has granted bioRxiv a license to display the preprint in perpetuity. It is made available under aCC-BY 4.0 International license.

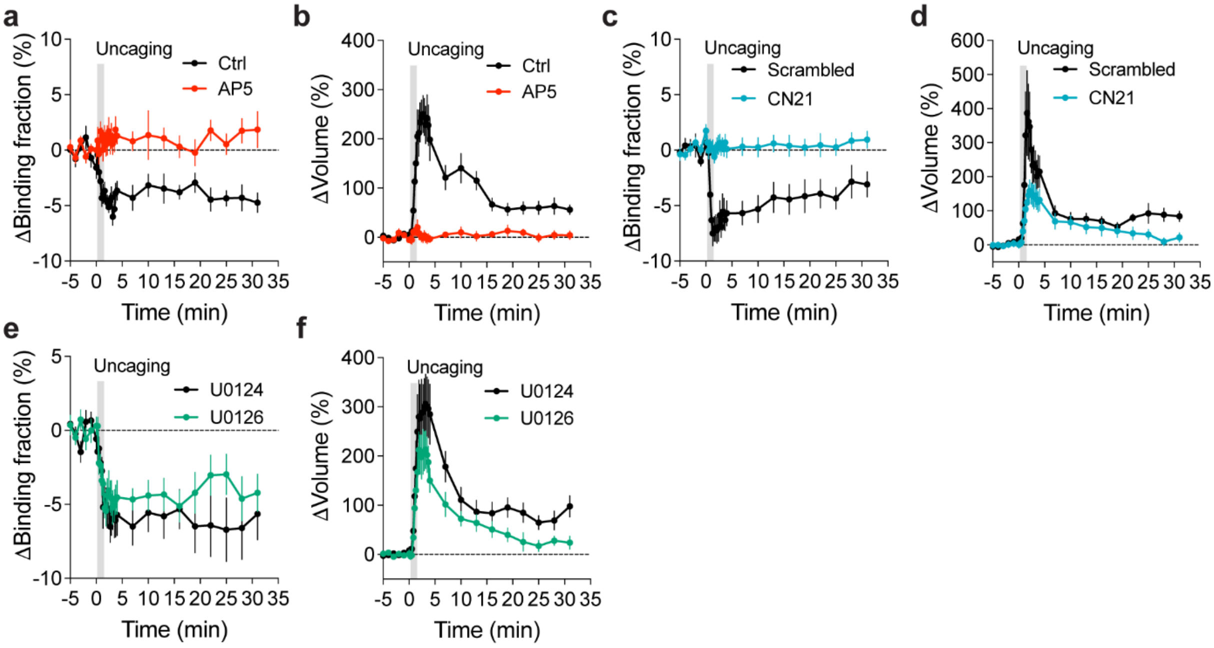

Figure 2-figure supplement 6 | Rab10 inactivation under manipulations of putative upstream signaling pathways.

(a and b) Averaged time courses for changes in binding fraction of Rab10 sensor (a) and volume (b) of the stimulated spines during sLTP. Black and red curves represent control (Ctrl) and AP5 (50 μM), respectively. Data represent mean ± SEM. N=15/14 and 13/11 (spine/neuron) for Ctrl and AP5, respectively. **(c and d)** Averaged time courses for changes in binding fraction of Rab10 sensor (c) and volume (d) of the stimulated spines during sLTP. Black and blue curves represent scrambled peptide control (10 μM) and CN21 peptide (10 μM), respectively. Data represent mean ± SEM. N=8/7 and 15/12 (spine/neuron) for scrambled peptide and CN21 peptide, respectively. **(e and f)** Averaged time courses for changes in binding fraction of Rab10 sensor (e) and volume (f) of the stimulated spines during sLTP. Black and green curves represent U0124 control (20 μM) and U0126 (20 μM), respectively. Data represent mean ± SEM. N=9/8 and 11/8 (spine/neuron) for U0124 and U0126, respectively. For all pharmacological inhibition experiments, hippocampal slices were incubated in the indicated drugs for 30 min before experiments. All experiments were paired with controls from neurons in the same batch of slices.

bioRxiv preprint doi: https://doi.org/10.1101/2022.05.17.492345; this version posted August 28, 2025. The copyright holder for this preprint (which was not certified by peer review) is the author/funder, who has granted bioRxiv a license to display the preprint in perpetuity. It is made available under aCC-BY 4.0 International license.

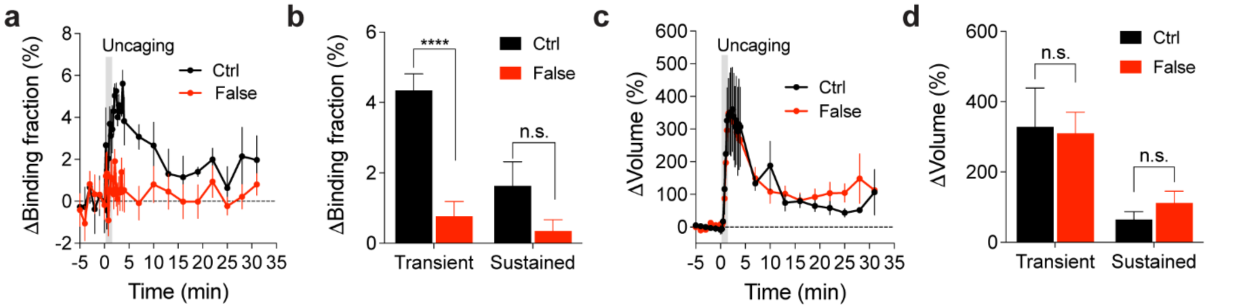

Figure 3-figure supplement 1 | Changes in the binding fraction of mEGFP-Rab4 paired with false acceptor during sLTP.

(a) Averaged time course of changes in binding fraction of Rab4 sensor (Ctrl, black) in the stimulated spines during sLTP. When mEGFP-Rab4 was paired with a false acceptor (False, red), mCherry-Rim1 [20-227]-mCherry, little activity change was observed. Data represent mean ± SEM. N=6/5 (spine/neuron) for Ctrl, and 12/9 for False. **(b)** Quantification of changes in binding fraction in the transient phase (1.3-4 min) and sustained phase (19-31 min) for the same experiment as in (a). Data represent mean ± SEM. Stars denote statistical significance (n.s., not significant, **** $p<0.0001$, Student's t-tests). **(c)** Averaged time courses of spine volume changes for the same experiments as in (a). **(d)** Quantification of changes in spine volume in the transient phase (1.3-4 min) and sustained phase (19-31 min) for the same experiments as in (a). Data represent mean ± SEM (n.s., not significant, Student's t-tests). Please note the quantification data in (b) and (d) are also presented in Figure 3c,d,f,g.

bioRxiv preprint doi: https://doi.org/10.1101/2022.05.17.492345; this version posted August 28, 2025. The copyright holder for this preprint (which was not certified by peer review) is the author/funder, who has granted bioRxiv a license to display the preprint in perpetuity. It is made available under aCC-BY 4.0 International license.

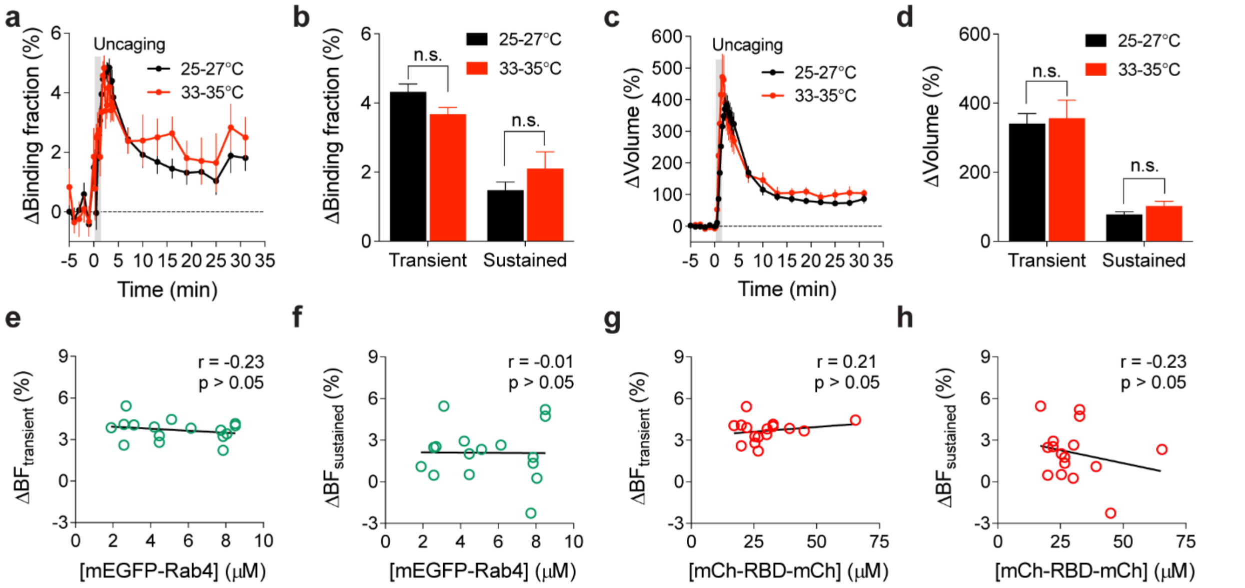

Figure 3-figure supplement 2 | Activation of Rab4 during sLTP induction at near physiological temperature.

(a) Averaged time courses for changes in binding fraction of Rab4 sensor in the stimulated spines during sLTP at 25-27°C (black) and 33-35°C (red). Data represent mean ± SEM. N=42/34 and 16/12 (spine/neuron) for 25-27°C and 33-35°C, respectively. **(b)** Quantification of binding fraction changes in the transient phase (1.3-4 min) and sustained phase (19-31 min) for the same experiments as in (a). Data represent mean ± SEM (n.s., not significant, Student's t-tests). **(c)** Averaged time courses of changes in spine volume for the same experiments as in (a). Data represent mean ± SEM. **(d)** Quantification of spine volume changes during the transient phase (1.3-4 min) and sustained phase (19-31 min) for the same experiments as in (a). Data represent mean ± SEM (n.s., not significant, Student's t-tests). Please note that the 25-27°C (black) samples in (a-d) are the same as those in Figure 3b, e. (**e and f**) Relationship between mEGFP-Rab4 concentration and changes in binding fraction of Rab4 sensor during the transient (e, 1.3-4 min) and sustained phase (f, 19-31 min) of sLTP at 33-35°C. No significant correlation ($p > 0.05$) was found. N=16/12 (spine/neuron). **(g and h)** Relationship between mCherry-RBD-mCherry concentration and binding fraction changes during the transient (g, 1.3-4 min) and sustained phase (h, 19-31 min) of sLTP at 33-35°C. No significant correlation ($p > 0.05$) was found. N=16/12 (spine/neuron).

bioRxiv preprint doi: https://doi.org/10.1101/2022.05.17.492345; this version posted August 28, 2025. The copyright holder for this preprint (which was not certified by peer review) is the author/funder, who has granted bioRxiv a license to display the preprint in perpetuity. It is made available under aCC-BY 4.0 International license.

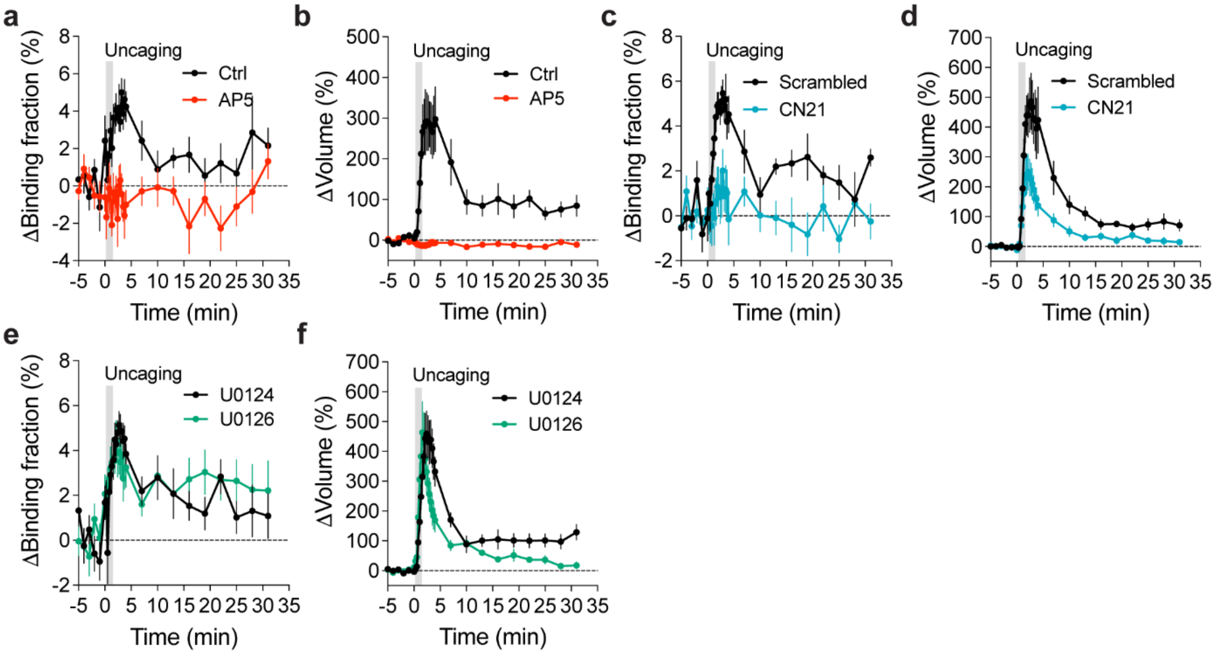

Figure 3-figure supplement 3 | Pharmacological evaluations of upstream signaling pathways mediating Rab4 activation.

(a and b) Averaged time courses for changes in binding fraction (a) of Rab4 sensor and volume (b) of the stimulated spines during sLTP. Black and red curves represent control (Ctrl) and AP5 (50 μM), respectively. Data represent mean ± SEM. N=8/6 and 12/9 (spine/neuron) for Ctrl and AP5, respectively. **(c and d)** Averaged time courses for changes in binding fraction (c) of Rab4 sensor and volume (d) of the stimulated spines during sLTP. Black and blue curves represent scrambled peptide control (10 μM) and CN21 peptide (10 μM), respectively. Data represent mean ± SEM. N=9/8 and 13/11 (spine/neuron) for scrambled peptide and CN21 peptide, respectively. **(e and f)** Averaged time courses for changes in binding fraction of Rab4 sensor (e) and volume (f) of the stimulated spines during sLTP. Black and green curves represent U0124 control (20 μM) and U0126 (20 μM), respectively. Data represent mean ± SEM. N=10/8 and 9/8 (spine/neuron) for U0124 and U0126, respectively. For all pharmacology experiments, hippocampal slices were incubated with the indicated drugs for 30 min before experiments. All experiments were paired with control neurons from the same batch of slices.

bioRxiv preprint doi: https://doi.org/10.1101/2022.05.17.492345; this version posted August 28, 2025. The copyright holder for this preprint (which was not certified by peer review) is the author/funder, who has granted bioRxiv a license to display the preprint in perpetuity. It is made available under aCC-BY 4.0 International license.

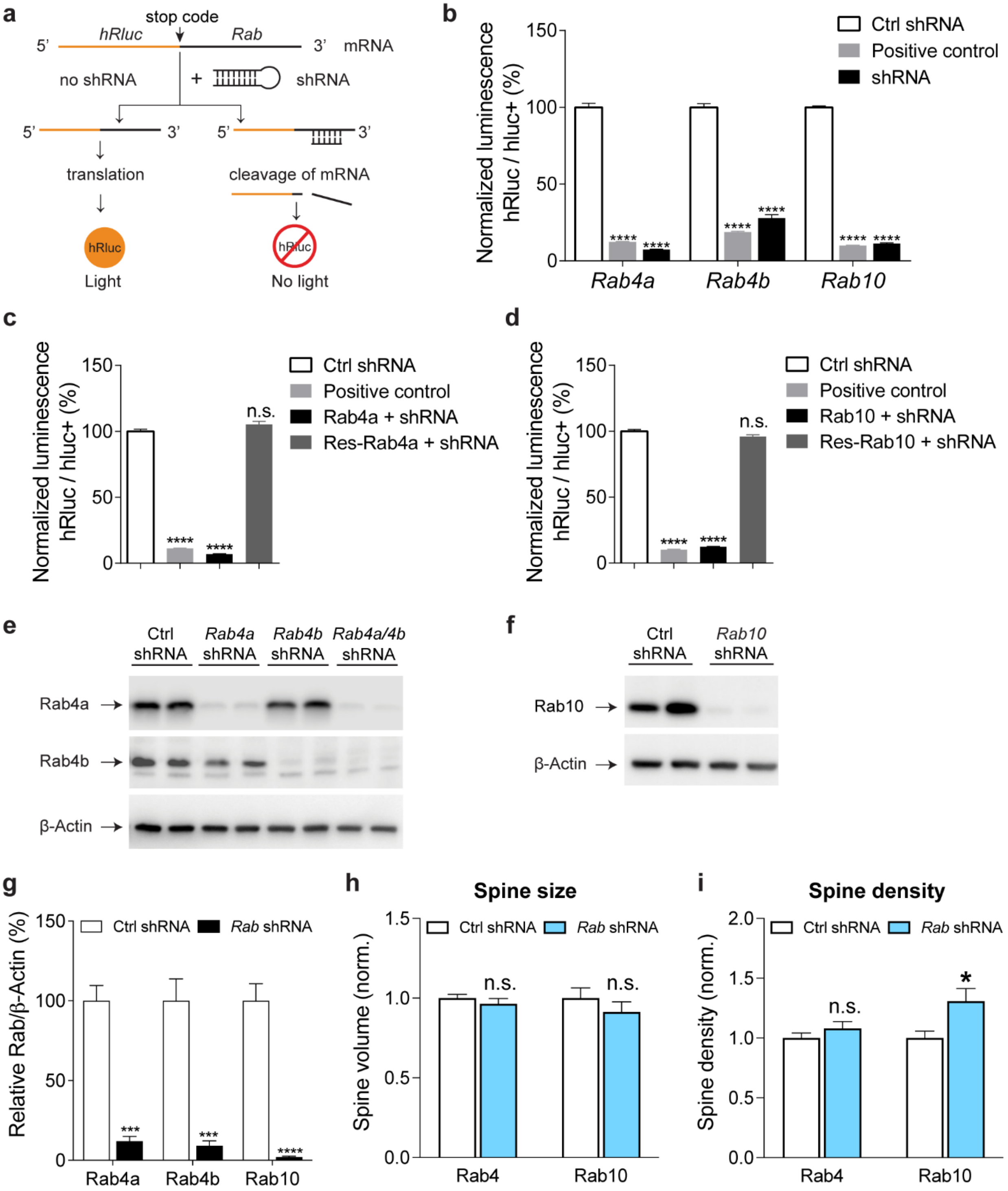

Figure 4-figure supplement 1 | Validation of *Rab GTPase* shRNA and shRNA-resistant Rab GTPases, and effects of Rab knockdown on spine size and density. **(a)** Schematic of dual-luciferase reporter assay. **(b)** Validation of knockdown by specific *Rab4a*, *Rab4b* and *Rab10* shRNAs using dual-luciferase reporter assay. Individual psiCHECK-2-Rab GTPase was cotransfected into HEK 293T cells with scrambled shRNA (Ctrl shRNA), *hRluc* shRNA (Positive control) or individual *Rab GTPase* shRNA. Data

bioRxiv preprint doi: https://doi.org/10.1101/2022.05.17.492345; this version posted August 28, 2025. The copyright holder for this preprint (which was not certified by peer review) is the author/funder, who has granted bioRxiv a license to display the preprint in perpetuity. It is made available under aCC-BY 4.0 International license.

represent mean ± SEM (**** $p<0.0001$, one-way ANOVA followed by Bonferroni's multiple comparison tests). N=8, 8, 8, 8, 8, 8, 10, 10, 10 wells from left to right. **(c)** Verification of shRNA-resistant Rab4a. HEK 293T cells were transfected with psiCHECK-2-shRNA-resistant Rab4a and scrambled shRNA (Ctrl shRNA), psiCHECK-2-shRNA-resistant Rab4a and *hRluc* shRNA (Positive control), psiCHECK-2 Rab4a and *Rab4a* shRNA (Rab4a+shRNA), or psiCHECK-2-shRNA-resistant Rab4a and *Rab4a* shRNA (Res-Rab4a+shRNA). Data represent mean ± SEM (n.s., not significant, **** $p<0.0001$, one-way ANOVA followed by Bonferroni's multiple comparison tests). N=4, 4, 4, 4 wells from left to right. **(d)** Verification of shRNA-resistant Rab10. HEK 293T cells were transfected with psiCHECK-2-shRNA-resistant Rab10 and scrambled shRNA (Ctrl shRNA), psiCHECK-2-shRNA-resistant Rab10 and *hRluc* shRNA (Positive control), psiCHECK-2 Rab10 and *Rab10* shRNA (Rab10+shRNA), or psiCHECK-2-shRNA-resistant Rab10 and *Rab10* shRNA (Res-Rab10+shRNA). Data represent mean ± SEM (n.s., not significant, **** $p<0.0001$, one-way ANOVA followed by Bonferroni's multiple comparison tests). N=5, 5, 5, 5 wells from left to right. **(e and f)** Validation of shRNA by western blot. Western blot of total protein extracts from cortical neurons cultured 15-17 days *in vitro* infected with lentiviral vectors expressing mEGFP plus either scrambled control shRNA or *Rab* shRNA. Data shown are representative of two independent experiments (n=4). **(g)** Quantification of the western blot experiments in (e) and (f). Data represent mean ± SEM (*** $p<0.001$, **** $p<0.0001$, Student's t-tests). **(h)** Knockdown of Rab4a/4b or Rab10 had no effect on basal spine size. Data represent mean ± SEM (n.s., not significant, Student's t-tests). N=20, 24, 20 and 20 (neurons) from left to right. **(i)** Knockdown of Rab4a/4b had no effect on spine density while knockdown of Rab10 enhanced spine density. Data represent mean ± SEM (n.s., not significant, * $p<0.05$, Student's t-tests). N=20, 24, 20 and 20 (neurons) from left to right.

bioRxiv preprint doi: https://doi.org/10.1101/2022.05.17.492345; this version posted August 28, 2025. The copyright holder for this preprint (which was not certified by peer review) is the author/funder, who has granted bioRxiv a license to display the preprint in perpetuity. It is made available under aCC-BY 4.0 International license.

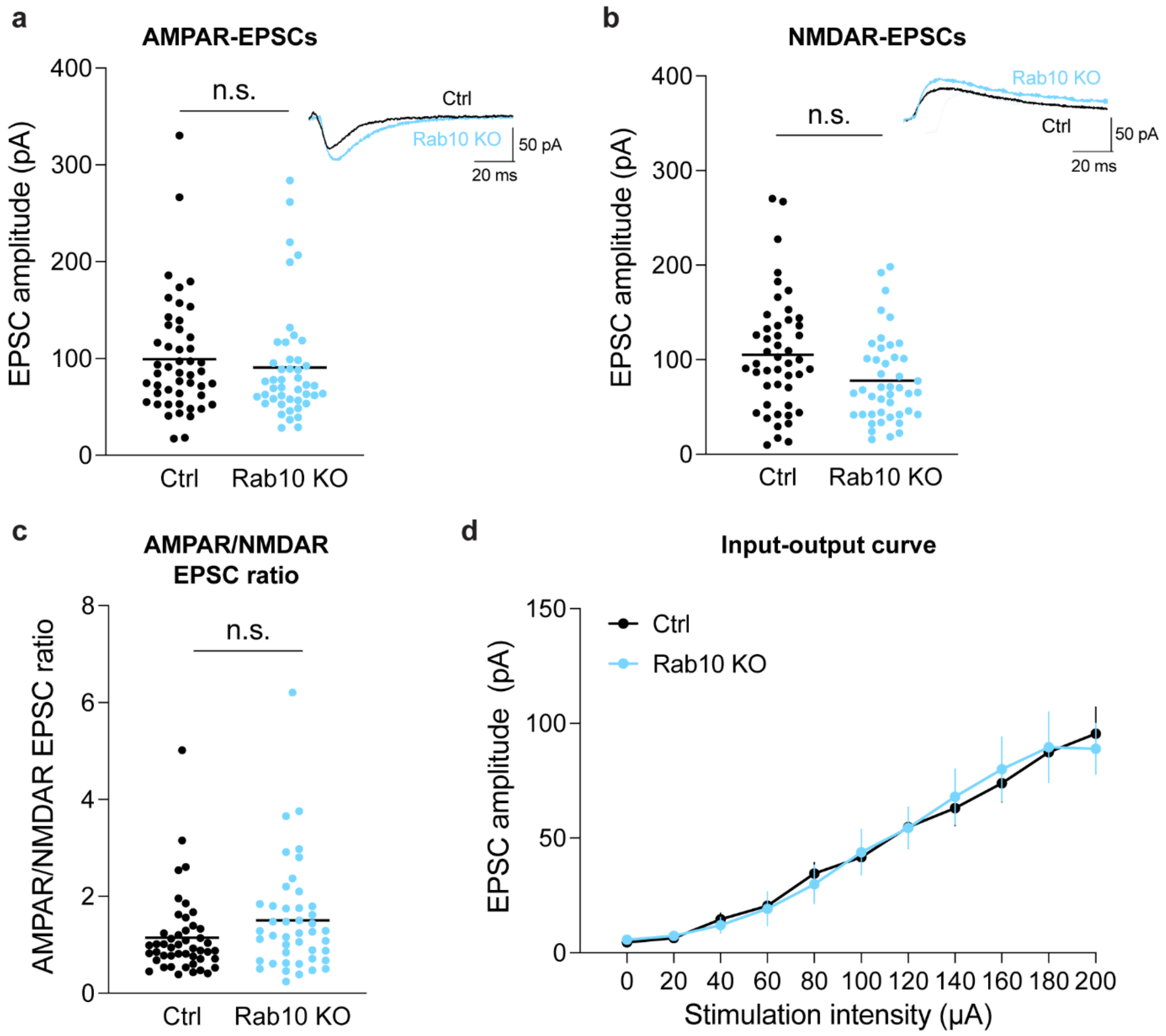

Figure 4-figure supplement 2 | Deletion of Rab10 has no effect on CA3-CA1 synaptic transmission in the hippocampus.

(a and b) Whole cell recordings of AMPAR (a) and NMDAR (b) mediated EPSCs in *Rab10*$^{fl/fl}$:*Camk2a-Cre*$^{+/-}$ (Rab10 KO) mice and littermate *Rab10*$^{fl/fl}$:*Camk2a-Cre*$^{-/-}$ control (Ctrl) mice. **(a)** Amplitude quantification of AMPAR EPSCs (at -70mV) in Ctrl (black, n=48/5 cells/animals) and Rab10 KO (blue, n=45/5 cells/animals) mice. Insets are representative evoked responses (black for Ctrl and blue for Rab10 KO). **(b)** Amplitude quantification of NMDAR EPSCs (at +40mV) for the same experiments in (a). To avoid contamination with residual AMPAR currents, the amplitude of NMDAR EPSCs was calculated by measuring the response amplitude at 50 ms after the peak. Insets are representative evoked responses. **(c)** AMPAR/NMDAR EPSC ratio for Ctrl (black) and Rab10 KO (blue) mice. **(d)** Input-output relationship for Ctrl (black) and Rab10 KO (blue) mice. No significant difference was detected in any of the results using unpaired t-tests.

bioRxiv preprint doi: https://doi.org/10.1101/2022.05.17.492345; this version posted August 28, 2025. The copyright holder for this preprint (which was not certified by peer review) is the author/funder, who has granted bioRxiv a license to display the preprint in perpetuity. It is made available under aCC-BY 4.0 International license.

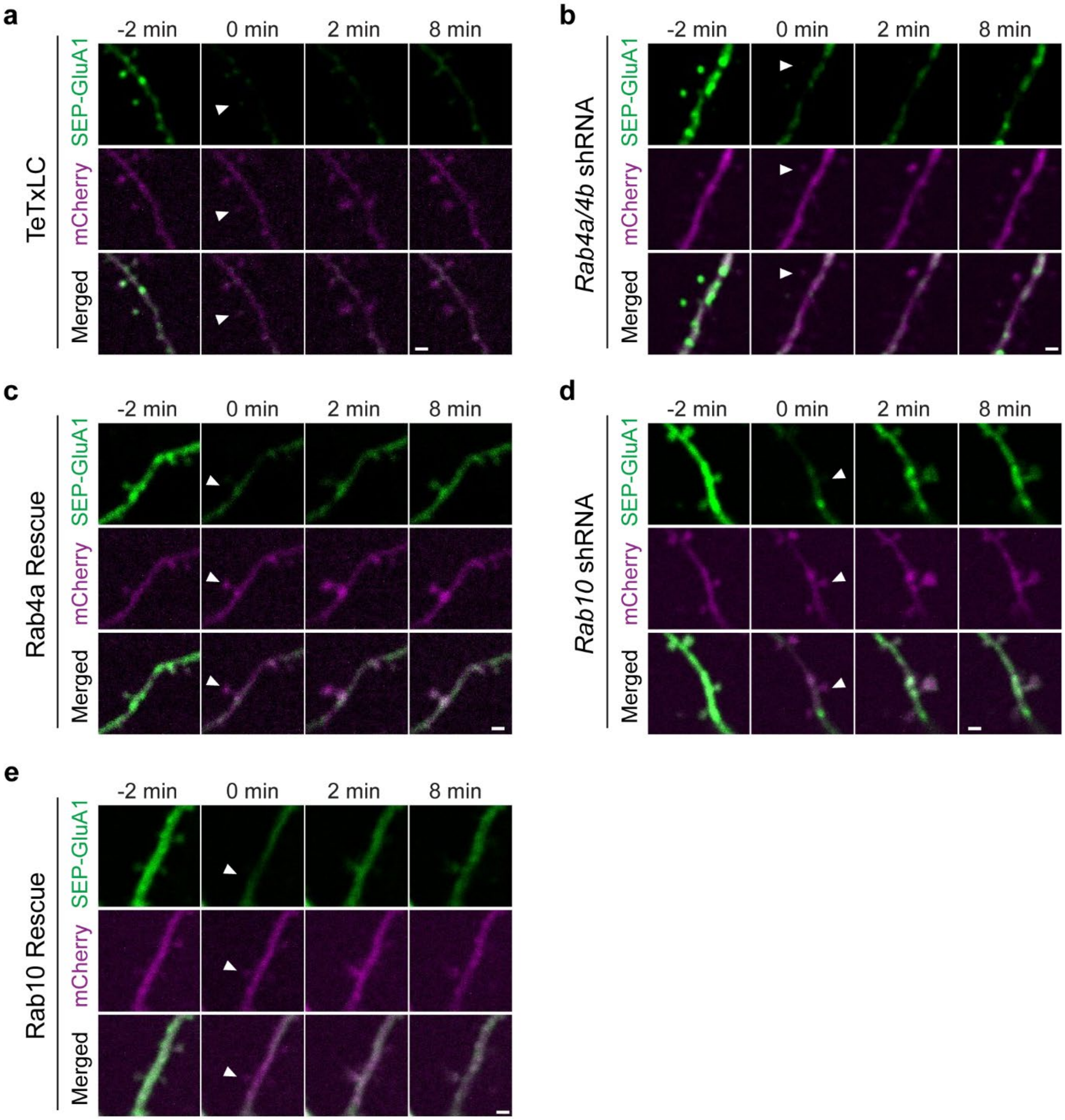

Figure 5-figure supplement 1 | Rab4 and Rab10 regulate activity-dependent GluA1 exocytosis in the stimulated spines during sLTP.

(a-e) Representative images of SEP-GluA1 (green) FRAP after two-photon glutamate uncaging in the stimulated spines of hippocampal neurons coexpressing mCherry and TeTxLC (a); mCherry and *Rab4a* and *Rab4b* shRNAs (b); mCherry, *Rab4a* and *Rab4b* shRNAs and shRNA-resistant Rab4a (c); mCherry and *Rab10* shRNA (d) or mCherry, *Rab10* shRNA and shRNA-resistant Rab10 (e). White arrowheads indicate the stimulated spine. Scale bar represents 1 μm.

bioRxiv preprint doi: https://doi.org/10.1101/2022.05.17.492345; this version posted August 28, 2025. The copyright holder for this preprint (which was not certified by peer review) is the author/funder, who has granted bioRxiv a license to display the preprint in perpetuity. It is made available under aCC-BY 4.0 International license.

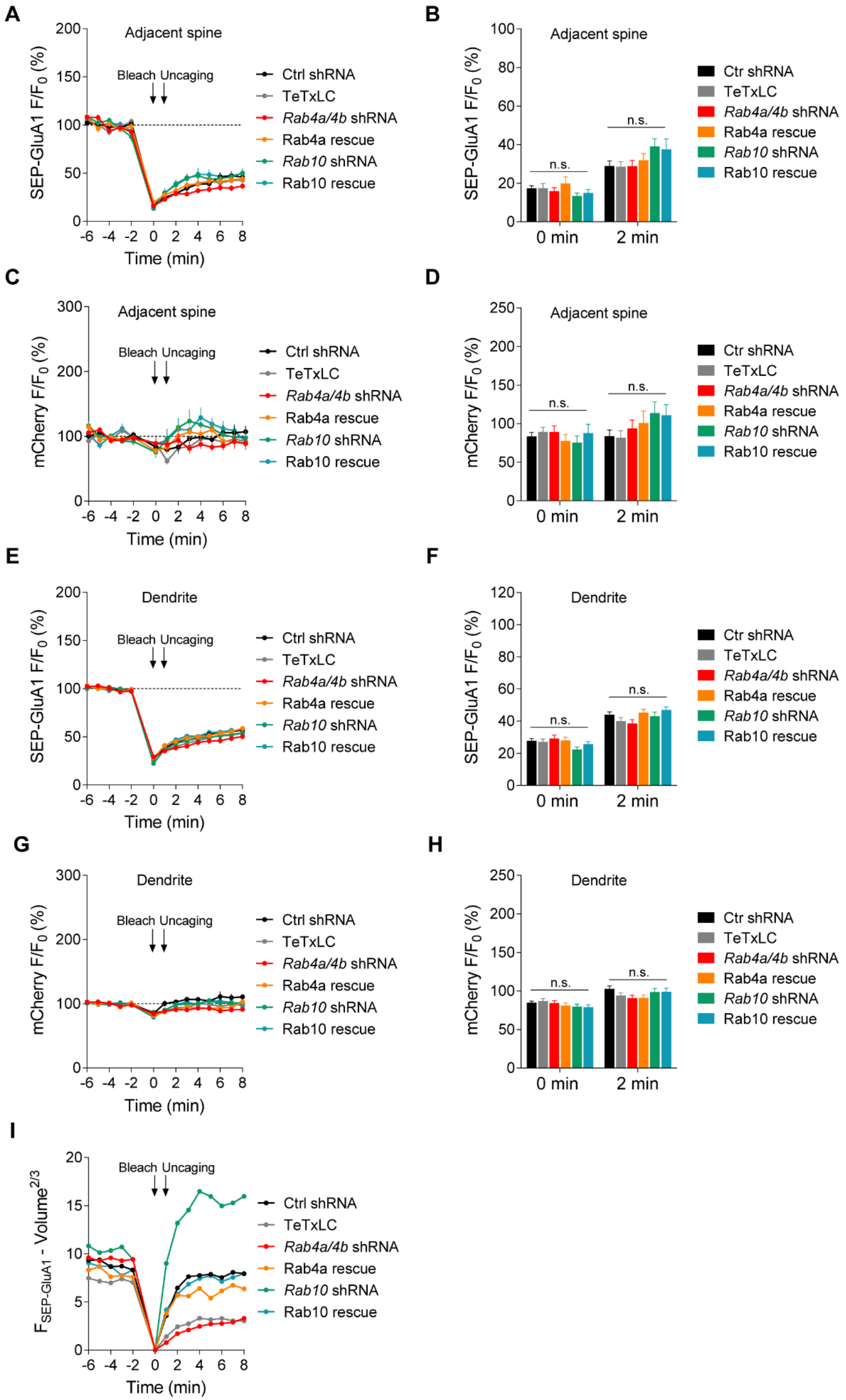

bioRxiv preprint doi: https://doi.org/10.1101/2022.05.17.492345; this version posted August 28, 2025. The copyright holder for this preprint (which was not certified by peer review) is the author/funder, who has granted bioRxiv a license to display the preprint in perpetuity. It is made available under aCC-BY 4.0 International license.

Figure 5-figure supplement 2 | Fluorescence intensity of SEP-GluA1 and mCherry in adjacent spines and dendrites.

(a and c) Averaged time courses of SEP-GluA1 (a) and mCherry (c) fluorescence intensity for adjacent spines in the same experiments as in Figure 5c. Data represent mean ± SEM. N=43/35, 19/12, 26/16, 16/12, 23/15 and 22/19 (spine/neuron) for Ctrl, TeTxLC, *Rab4a/4b* shRNA, Rab4a rescue, *Rab10* shRNA and Rab10 rescue, respectively. **(b and d)** Quantification of SEP-GluA1 (b) and mCherry (d) fluorescence intensity for adjacent spines at 0 min and 2 min. Data represent mean ± SEM (n.s., not significant, one-way ANOVA followed by Bonferroni's multiple comparison tests). N=43/35, 19/12, 26/16, 16/12, 23/15 and 22/19 (spine/neuron) for Ctrl, TeTxLC, *Rab4a/4b* shRNA, Rab4a rescue, *Rab10* shRNA and Rab10 rescue, respectively. **(e and g)** Averaged time courses of SEP-GluA1 (e) and mCherry (g) fluorescence intensity for dendrites in the same experiments as in Figure 5c. Data represent mean ± SEM. N=43/35, 19/12, 26/16, 16/12, 23/15 and 22/19 (dendrite/neuron) for Ctrl, TeTxLC, *Rab4a/4b* shRNA, Rab4a rescue, *Rab10* shRNA and Rab10 rescue, respectively. **(f and h)** Quantification of SEP-GluA1 (f) and mCherry (h) fluorescence intensity for dendrites at 0 min and 2 min. Data represent mean ± SEM (n.s., not significant, one-way ANOVA followed by Bonferroni's multiple comparison tests). N=43/35, 19/12, 26/16, 16/12, 23/15 and 22/19 (spine/neuron) for Ctrl, TeTxLC, *Rab4a/4b* shRNA, Rab4a rescue, *Rab10* shRNA and Rab10 rescue, respectively. **(i)** SEP-GluA1 FRAP after subtraction of the surface area increase in the stimulated spines for experiments in Figure 5c. N=43/35, 19/12, 26/16, 16/12, 23/15 and 22/19 (spine/neuron) for Ctrl, TeTxLC, *Rab4a/4b* shRNA, Rab4a rescue, *Rab10* shRNA and Rab10 rescue, respectively.
